# 

**Published:** 1891-07-25

**Authors:** 


### NOTICE TO CORRESPONDENTS.

All MS., Letters, Books for Review, and other matters intended for the Editor should be addressed The Editor, The Lodge, Porchester Square, London, W.

The Editor cannot undertake to return rejected M.S., even when accompanied by stamped directed envelope.

### Notice to Advertisers and Subscribers.

All Advertisements, Orders for Copies of The Hospital, and Business Communications should be addressed to The Publisher, *not to the Editor*, The Hospital, 140, Strand, W.C.

The Cost of Subscription, post free, is as follows:—Per week, 1½d.; per quarter, 1s. 8d.; per half-year, 3s. 3d.; per annum, 6s. 6d. Foreign:—Per annum, 8s. 8d.; payable, in all cases, in advance.

**NOTICE.—The Index for Vol. IX.. (ending March, 1891) is on sale at the Office of this Journal, price 2½d., post free.**

### GENERAL CHARITIES.

[This Column is devoted to an account of work of charitable institutions other than Medical Charities. The Editor will be glad to receive reports or news of work at 140, Strand. *Philanthropy* should be plainly written on the envelope.]

The new mission house given by Miss Catherine Phillimore to the **CHRIST CHURCH (OXFORD) MISSION** at the East India Docks is to be the resting place of a community of Clewer Sisters. The Mission has now been doing its good and unostentatious work for ten years.

Among the contributions recently received by the **ROYAL NATIONAL LIFEBOAT INSTITUTION** are £700 from Mr. and Mrs. Norbury's lifeboat fund for the Porth Rhuffyd new lifeboat; £700 from the executors of the late Mrs. Margaret Platt for the new lifeboat for Rhyl; £200 from the Ancient Order of Foresters; £100 from Miss Lucy Harris, in aid of the maintenance of her lifeboat at Plymouth, the "Escape"; £10 collected on board the steamship "Orotava"; and £4 5s. 6d. collected on board the "St. Sunniva"; making in all £1,700 5s. 6d.

A fresh departure is being taken by the **HARROW MISSION**, which is practically a natural development of its work. The sum required to carry out the extension is £5,000, and the object of the new scheme is to provide men's and boys' clubs at the mission in Latimer Road, Notting Hill. As we pointed out last week there are many thickly-populated parts of the West-end where there is quite as much poverty and misery crying out to be alleviated as in the East-end, and it is to such localities that the Mission pays attention. Since the opening of the permanent Mission House in 1884 a church has been built, and it is hoped that the carrying out of the present scheme will serve to consolidate the work of the mission.

After much deliberation, it has been definitely decided to establish a **JOURNALISTIC ORPHAN FUND**, the report of the Special Committee of the Council of the Institute of Journalists to consider the question of establishing orphan and benefit funds having been adopted last week so far as it relates to an orphan fund. Sir Algernon Borthwick, who is President of the Newspaper Press Fund, has promised £1,000, and £500 have been promised by Mr. J. A. Willox, of the *Liverpool Courier*. We are given to understand that the Fund will be administered by the Institute, but the necessity for such a fund does not seem very clear, and its establishment appears to be a flagrant instance of the multiplication of charities, and consequent waste of money and strength. The machinery for distributing such a fund already exists in the Newspaper Press Fund, where, judging from the fact that the management are able annually to lay by on the average about £1,000, there are ample means for providing for orphans of journalists who come within the scope of this Society.

An interesting ceremony took place recently in Bermondsey, when the Lord Mayor laid the foundation-stone of an institution which is to be devoted to the help of the young when they leave school at a critical time—a time when children need a kind, fostering, and watching hand. The **BERMONDSEY SETTLEMENT** in Farncombe Street is the outcome of a scheme adopted recently by Wesleyan members of the Universities of Oxford, Cambridge, Edinburgh, and London. The institution will be associated with numerous schools in different parts of the country, and the building in Farncombe Street, which is to cost £13,500, will contain a students' library and reading-room, class-rooms, a lecture hall, a chemical laboratory, a workshop, a gymnasium, a warden's house, and residence for about twenty men, and a room fitted with apparatus for cookery and dressmaking. The combination of cookery and dressmaking sounds incongruous and unpleasant. All the classes will be open to both sexes, and the educational work will include the delivery of University Extension lectures, occasional popular lectures, choral and orchestral societies, and classes in the different branches of science, literature, and art.

According to the last report of the **SOLDIERS' DAUGHTERS HOME, HAMPSTEAD**, the finances of the institution have never before attained such a degree of prosperity as they have now reached. Its position in this respect will be a matter of envy to many other societies, who, however, are sometimes apt to forget that a balance on the wrong side of their banker's book is occasionally due, not so much to want of generosity on the part of the public as to extravagant and uncalled-for expenditure. The Home at Hampstead is for the maintenance, clothing, and education of the daughters of soldiers, whether orphans or not, and is supported very generally by contributions from the army, though the names of far too many regiments are conspicuous by their absence from the list of subscribers. If every regiment did only half of what is done by the Royal Engineers, who support nine boarders in the Home, there would be no fear as to maintaining the present prosperous condition of the institution. There are now 191 children in the Home, every one of whom was present at the anniversary meeting last month, not one of them being at the time in the infirmary, which speaks well for the care taken of them. A satisfactory feature in the report is the reduction in the cost of maintenance. The average number of girls in the Home during the past year was 177, and the average cost of maintenance per head was £20 8s. 5½d., being 17s. 2½d. less than in the previous year.

### SCRAPS AND GLEANINGS.

THE liquor bill at the Edinburgh Fever Hospital still continues to create a good deal of excitement.

WE hear that Dr. Tibbits' official connection with the West End Hospital ceased at the beginning of this month.

THE Rev. Professor Marks, of the West London Synagogue for British Jews, has sent £216 to the Hospital Sunday Fund.

SIR SYDNEY AND LADY WATERLOW gave a concert on the 20th inst. to the nurses on the staff of St. Bartholomew's Hospital.

ON the 10th inst. the New Hospital at Sunny Side, N.B., was formally inaugurated. It has been erected at the cost of £140 per bed.

THE Worshipful Company of Clothworkers have given a donation of ten guineas towards the funds of the Gardeners' Royal Benevolent Institution.

A SALE of work and concert were held at Blackrock in aid of the Cork Hospital for Women and Children, when a substantial sum was handed over to the Treasurer.

IT is stated that the weekly contributions of working men have formed over one-third of the whole income of the Tynemouth Victoria Jubilee Infirmary during the past year.

THE West London Hospital is now in possession of an ample building site for the much-needed extension of the institution. At present it is an ordinary event to refuse as many as eight suitable cases daily for want of space.

THE annual meeting of the Friendly and Trade Societies at Oxford, in aid of the medical charities of the city, took place on Tuesday, 7th inst. The sum collected amounted to £98, a smaller result than that obtained the previous year.

THE financial condition of the North-west London Hospital gives cause for grave anxiety. The annual subscriptions at present yield only £800, towards a yearly expenditure of £3,500. There are large outstanding liabilities.

THE Duke of Cambridge will on Monday, the 27th inst., formally open the Surrey Convalescent Home at Seaford, of which he is president. The building has been erected by an anonymous donor, and has cost £25,000, including the endowment.

THE ninth annual report of the Hampstead Home Hospital and Nursing Institute shows a steady increase in the usefulness of the hospital, and proceeds of the festival dinner have proved sufficient to clear off the debt existing at the beginning of the year.

AT the annual meeting of the Oldham Hospital Saturday Committee, Mr. Ingham made an appeal on behalf of the Oldham Infirmary, which shows a deficit of £781 on its accounts. At the present time 642 in-patients and 1,845 out-patients are treated annually.

IN recognition of the pressing needs of the West London Hospital, in the Hammersmith Road, the Goldsmiths' Company have recently voted in aid of its funds £100, the Grocers' Company £50, the Girdlers' Company £26 5s., and the Vintners' Company £10 10s.

THE thirty-second annual report of the Pembrokeshire and Haverfordwest Infirmary shows a deficiency of £119 on the accounts. The Matron (Miss Bull) is to be congratulated on the able manner in which she conducts the internal management of the Infirmary.

THE annual meeting and distribution of prizes of the Association for the Oral Instruction of the Deaf and Dumb was held during the week at 11, Fitzroy Square. During last year 36 boys and 14 girls attended the Society's school, while seven teachers received training, namely, six females and one male.

THE Committee of the New General Hospital at Birmingham have appointed Mr. Waterhouse, R.A., as consulting architect for the building of the new hospital. They have also considered, but have not yet finally decided, the terms of the limited competition for designs and plans, and the list of architects who are to be invited to take part in the competition.

THE Lady Mayoress distributed prizes on 11th inst. at Aske's Hatcham School for Girls in the new school building, Jerningham Road, New Cross. The school is controlled by the Haberdashers' Company, and shows numerous successes in the examinations of the Science and Art Department, the Apothecaries' Company, and in the Cambridge Local Examinations.

THE fifteenth annual report of the Cheyne Hospital for Children shows that the double capacity of the building has not caused a proportionate increase in the expenditure, a fact upon which the Committee are to be congratulated, as the average expenditure of this hospital has never been a high one. Several legacies of considerable amount have fallen to the lot of the institution.

LADY WOLVERTON, the founder and President of the Houseboys' Brigade, recently distributed the prizes at a supper given to the "old boys of the institution," at St. Mary's School, Bryanston Square. The brigade has now attained its majority. Since its formation over 500 boys have passed through the home, where they have been trained for domestic service and other occupations.

THE Committee of Management of the Clinical Hospital for Women and Children, situated in Park Place, Cheetham, have purchased two large houses in York Street, and improvements in the existing establishment are to be made. The cost of the new portion and the alterations will be about £2,000, and towards this about £1,700 has been contributed by friends and patrons.

ONE THOUSAND AND FORTY boxes, placed mainly in the East Central and West Central Postal Districts on Hospital Saturday, contained £1,650, of which £179 was in gold, two and a-half cwt. in silver, and two tons in bronze money. A comparison between this and last year is a little deceptive, owing to a rearrangement of the districts, and we hope the aggregate result will show an improvement.

THE Hospital for Epilepsy and Paralysis is fortunate in its friends. For several years its revenue has been substantially increased by the proceeds of a series of entertainments held in its behalf, and now an anonymous donor has come forward with a gift of £1,200, which has placed the institution rent free for ten years. The Committee have decided to raise a building fund for a new hospital containing at least 50 beds.

### The Student of Medicine.

[All communications for this Supplement should be addressed to The Editor of The Hospital, at 140, Strand, with the words, "Students' Column," written in the left hand top corner of the envelope.]

### EDITORIAL NOTES.

The list of the June Matriculation of the London University has been published. The honour division is headed by Miss E. Charlotte Higgins, a student of the Edinburgh Association and the Royal Holloway College, and she has obtained the exhibition of £30 a-year for two years. All the other exhibitions and prizes have been gained by men. There were just upon 1,700 candidates, and of these 888 passed, that is, rather over 50 per cent. Of the candidates who have satisfied the examiners, 77 have gained honours, and 13 of these are women. In the first division there are 664 names, and in the second division 147.

The *Manchester Courier* has received from its "special correspondent" in New York a rather extraordinary story. A man in Tennessee was three times lynched for horse-stealing, but on each occasion, after the lynchers had left, he cut himself down, and made off. The natives considered he had a charmed or supernatural life. The secret of his escape, however, lay in the fact that he had inserted a silver tracheotomy tube into his larynx, and hence prevented the compression of the air passage and suffocation. It appears, however, that the tube one day became dislodged, and he has died from suffocation.

### NOTES FROM THE SCHOOLS.

### Edinburgh University.

The E.U. Lawn Tennis Club have had another successful season, winning each of the seven team matches they have played.

The Edinburgh University Baseball Club claim the Scottish Challenge Club as their right has not been contested. During the vacation the team will tour in England.

The Committee of the E.U. Cycling Club have recommended the following to the E.U. Athletic Club for "blues": Full Blue—A. Mann and H. J. Pechell. Half Blue—E. P. Dickin.

### APPOINTMENTS.

At the London Hospital F. Charlewood Turner, M.A., M.D. Camb., F.R.C.P.Lond., M.R.C.S., has been appointed physician. At the Home and Infirmary for Sick Children and South London Dispensary for Women, Lower Sydenham, E. W. Du Buisson, M.R.C.S., L.R.C.P., has been appointed honorary assistant medical officer, and F. J. Lorimer Hart, M.B., C.M.Edin., honorary assistant surgeon. At the City of Dublin Hospital, Humphrey John Broomfield, L.R.C.P., L.R.C.S.Irel., has been appointed assistant physician, and Mr. Cheevers resident medical student. At the Norwich City Asylum, Hellesdon, E. Caudwell, M.R.C.S., L.R.C.P., has been appointed assistant medical officer. At the Monkwearmouth Hospital, Sunderland, Arthur Holdsworth Davis, M.B.Lond., M.R.C.S., L.R.C.P., has been appointed assistant surgeon. At the Brailes District of the Shipton-on-Stour Union, George Findlay, M.B., C.M.Aber., has been appointed medical officer. At the Hammerwich District and Cottage Hospital, near Walsall, William F. Haslam, F.R.C.S., has been appointed consulting Surgeon. At the Hants County Asylum, Knowle, Fareham, Francis O. Simpson, L.R.C.P., M.R.C.S., has been appointed third assistant medical officer.

### VACANCIES.

The following vacancies are announced this week, the amount of salary in each case being given after the name of the institution:

### Resident Medical Officers.

Chorlton-upon-Medlock Dispensary, Manchester, House Surgeon—Salary £100 and board, &c.
City of London Hospital for Diseases of the Chest, Victoria Park, E., House Physician—Board, &c.
Devon County Lunatic Asylum, Assistant Medical Officer—Salary £120 and board, &c.
Devon and Exeter Hospital, Two Resident Pupils.
General Hospital, Birmingham, three Assistant House Surgeons—Board, &c.
Great Northern Central Hospital, Holloway Road, N., House Physician—Salary £80 and board, &c.
Hospital for Sick Children, Great Ormond Street, Bloomsbury, two Casualty Medical Officers.

### The Hospital Advertiser.

### MEDICAL APPOINTMENTS.

### THE HOSPITAL FOR SICK CHILDREN,

Great Ormond Street, Bloomsbury.

### THE HOSPITAL FOR SICK CHILDREN,

Great Ormond Street, Bloomsbury.

### THE STUDENT OF MEDICINE—(*continued*).

Hospital for Women, Soho Square, W., House Physician—Salary £30 and board, &c.
Liverpool Dispensaries, Assistant Surgeon—Salary £80 and board, &c.
Metropolitan Asylums Board, Clinical Assistant for the South Eastern Fever Hospital, New Cross Road, S.E.—Board, &c.
Nottingham Borough Asylum, *locum tenens* for a month.
Royal Albert Edward Infirmary and Dispensary, Wigan, Senior House Surgeon—Salary £100 and board, &c.
Royal Hospital for Diseases of the Chest, City Road, E.C., Medical Officer—Salary £100 and board, &c.

**Non-Resident Medical Officers.**

Abbey Parochial Board, Paisley, Medical Officer for the Eastern District Salary £30 and fees.
Applecross Parochial Board, Medical Officer—Salary £95 and house, &c.
Brighton, Hove, and Preston Dispensary, Honorary Surgeon-Dentist for the Western Branch.
Chorlton-upon-Medlock Dispensary, Manchester, Honorary Medical Officer.
Cork Union, Medical Officer—Salary £125 and fees.
Hampstead Provident Dispensary, New Road, N.W., Medical Officer.
Hospital for Sick Children, Great Ormond Street, Assistant Physician.
London Hospital, Assistant Physician.
Manchester Royal Infirmary, Junior Administrator of Anæsthetics—Salary £50.
Parish of St. Leonard, Shored tch, Medical Officer of Health—Salary £200.
Parochial Board of Morvern, Argyllshire Medical Officer—Salary, £130.

**PASS LISTS.**

**Society of Apothecaries of London.**

The following candidates have passed the recent Primary Examinations in the subjects indicated :—

ANATOMY AND PHYSIOLOGY.—N. B. Baker, St. Bartholomew's Hospital; A. H. A. Boyle, E. T. Dodson. H. M. Greene, A. Harding, A. M. Weir, and W. A. Westlake, Royal Free Hospital; H. E. Davis, St. Thomas's Hospital; H. Exell, Sheffield Medical School; R. J. Farman, Charing Cross Hospital; P. D. Fethers. St. Mary's Hospital; L. F. C. Hughes, Yorkshire College, Leeds; and H. D. N. Mackenzie, Edinburgh University.

ANATOMY.—W. F. Campbell, King's College; W. R. Fisher, London Hospital; T. Homer, Edinburgh University; and S. Langton, St. Mary's Hospital.

PHYSIOLOGY.—C. Bayley and F. H. Humphris, Edinburgh University; S. Foster and A. W. Robertson, St. Bartholomew's Hospital; J. Garner, D. F. Maunsell, and W. Ranson, St. Thomas's Hospital; J. B. Hodgson, Manchester and Glasgow; E. S. Johnson and G. C. Schultz, St. Mary's Hospital; M. Martin, Sheffield Medical School; G. W. Pauli, Bristol Medical School; A. J. Pollard, Yorkshire College, Leeds; and M. N. H. Roberts, Middlesex Hospital.

CHEMISTRY, MATERIA MEDICA, BOTANY, AND PHARMACY.—R. Crawford, Belfast; C. T. Green, Rukhmabai, and L. E. V. Saville, Royal Free Hospital; J. C. McWalter, Dublin; and G. E. H. Sargent, London Hospital.

CHEMISTRY AND PHARMACY.—E. Bentham, C. A. S. Fitter, M. Harmar, and K. M. Hunter, Royal Free Hospital; and G. Steel, Cambridge University.

PHARMACY.—L. M. Blake and E. L. M tcheson, Royal Free Hospital; W. A. King, Charing Cross Hospital, and H. D. N. Mackenzie, Edinburgh University.

**Final M.B.C.M. Exam, Edinburgh.**

Willie Adam, Alex. S. Anderson, Alf. W. Anderson, Jas. Anderson, M.A., T. Anderson. R. J. Ashton, B.A., F. R. B. Atkinson, Jas. Atkinson, Louis G. Barbean, Jos. H. Bayley, Hugh Bennett, R. J. A. Berry, L.R.C.S. and P., Edinburgh, Alf. J. Biennsman, T. H. Bishop, H. Blundell, T. W. Bone, G. E. Bowker, J. J. Brennan, T. B. Brierley, J. D. Broadfoot, S. C. Brush, W. M, Cairns, K. M. Cameron, Dan Campbell-P. Capper, C. W. Chapman, H. A. Clemons, H. A. Clark, E. J. W. Carruthers, C. W. Cochrane, R. P. Cockburn, C. J. Van Coller, J. W. Compton, J. Cowan, R. Crane, J. H. Crawford, J. F. Crombie, H. W. Cross, G. D. Darlington, Jas. Davidson, M.A., H. R. L. Davies, R. G. Dempster, W. B. Drummond (distinction), A. Duke, M A., J. A. H. Duncan, S. Edgerley, M.A., George Elder (distinction) A. C. Elliot, Michael Ernin, F. W. Enrich, J. L. Fenton, B.A., L. G. Funk, E. C. Fischer, J. L. Fletcher, W. D. Forsyth, F. F Foster, E. J. Fox, John Francis, H. E. Fraser, M.A., C. Frier, E. B. Fuller, W. M. D. Gallic, D. Gibb, J. Glover, F. Gourlay, E. C. H. Grenier, W. Griffith, W. C. Grosvenor, M.A., H. B. Hall, A. A. S. Harris, S. H. Hartley, A. J. Haslam, P. J. Hatton, E. Hay, W. P. Hay, R. A. Heath, G. E. Helme, T. R Henderson, M.A., Geo. Hennan, S. Hillier, G. J. B. Hope, B.A., G. V. Hulme, R. B. Huxtable, P. R. W. Jaqaundham, B.A., H. S. W. Jones, B.Sc., A. B. Kenworthy, E. Kirmont, R. Lamb, M.A., L. J. Lamrock, E. P. T. von Landsberg, W. E. Lee, B.A., Cyril Lewis, B.A., John Lewis Jones, John Livrington, A. J. M'Cullum, J. M'Cleymont, T. F. Macdonald, W. M. Macgill, Wakefield Macgill, T. N. Macgowan, F. W. Mackay, J. J. Mackay, G. M'Kellar, D. J. Mackenzie, M.A., F. J. M'Ettrick, A. J. Mackintosh, Arch. Mackintosh, John Maclaren, Sam. Maclean, W. B. Macleod, Charles Macmather, D. Macmillan, M.A., J. L. Macrae, F. R. Mallet, C. Martin, T. G. Matthews, J. S. Maynard, G. Melville, W. Mill, B A., James Miller, T. H. Milroy, W. C. Milroy, M.A., G. H. Mitchell, E. C. Moore, L. P. More, P. St. C. More, F. W. Moss, J. D. R. Munro, J. A. Murison, W. Murray, A. J. Van Nickerk, B.A., S. I. Oddie, W. H. Ogilvie, John Orr, T. L. Parry, W. Peart-Thomas, G. W.

**THE STUDENT OF MEDICINE—(*continued*).**

Prance, R. L. Price, H. Rainy, M.A. (distinction), J. M. Renton, M.A., E. H. Ridley, J. H. Roberts, A. G. Robertson, J. F. Robertson, M.A., A. G. Robertson, F. A. Rodrignez, J. C. Rossie, A. Rutherford, A. Rutter, J. B. Scott, R. W. L. Scott, S. A. Shiach. E. W. Slavter, J. B. Smith, J. G. Smith, J. Spurway (distinction), E. M. Stevin, T. Stodart, A. Stodart-Walker. Charles Stuart, David Stuart, C. A. Sturrock, M.A., C. D. Sutherland, John Sutherland, W. S. Syme, G. Templeton (distinction), B. Thomas, W. Thyne, M.A., D. D. Tindal, W. S. Walker, T. D. Walker, B.A., W. J. Walker, B.A., D. H. Welsh, G. S. Walton, G. G. Watson, N. P. Watt, M.A., A. I. Webster, J. H. G. Whiteford, B.A., D. Wield, M.A., R. S. Wilde, J. C. Williams, J. D. Williams, J. W. Williams, Geo. Wilson, M.A., G. R. Wilson, J. T. Wilson, T. A. M. Wilson, J. M. Wood, D. Young, J. Y. S. Young, R. J. E. Young, and F. S. Zaytoun.

**Royal Colleges of Physicians and Surgeons, Ireland: Conjoint Scheme.**

The following have passed the First Professional Examination:—J. Beaumont, E. J. Bennett, R. Coffey, Thos. Coulon, T. J. Considine, J. J. Cormack, B. Coyle, G. F. Creighton, J. H. Davys, K. Delany, M. Delany, J. Dwyer, J. W. Griffin, J. Halligan, P. Heanen, T. J. Jordan, R. K. Joyce, H. T. Kennedy, E. F. L'Estrange, A. Leventon, James Lynch, D. R. Love, W. Marriott, H. Martin, J. Martin, E. Meeke, P. Morrissey, W. Murphy, C. O'Gorman, J. F. O'Keeffe, E. F. Power, J. E. Shera, R. E. Smith, J. F. Smyth, W. Shatton, M. Walsh, and F. W. Woods.

**Royal Colleges of Physicians and Surgeons of England.**

The following list of candidates passed the Second Examination of the Board at a meeting of the Examiners on the 6th inst.:—

Anatomy and Physiology.—Henry E. Firth, Raphael A. Lewty, Edmund Welch, and Lancelot B. Todd, students of Yorkshire College, Leeds; John A. Eatock and John R. Ambler, of University College, Liverpool; Edward A. Purcell and Alfred W. Joynson, of Dublin; Frederic E. Peake, of Bristol Medical School; Archibald Young, of Sheffield Medical School; G. Aubrey Jelly, Francis W. Hartley, and Arthur P. Shaw, of Owens College, Manchester; Herbert M. Pentreath, of St Bartholomew's Hospital; Nathaniel H. Hobart, of Cambridge University; and William T. Pugh, of Middlesex Hospital.

Anatomy Only.—Alfred R. Hitchfield, of Guy's Hospital; Gerald A. Child, of Oxford University; Lockhart Lowe and Karl F. Cherry, of Queen's College, Birmingham; Ernest Moore, of St. Thomas's Hospital and Mr. Cooke's School of Anatomy and Physiology; Isaac Watts, of Owens College, Manchester; John D. McD. Newlands and Francis W. Bailey, of University College; Edward Brabazon, of Dublin; Chas. J. P. T. Gibbons, of London Hospital; and Maurice C. Barber, of Bristol Medical School.

Successful on the 7th inst.:—

Anatomy and Physiology.—John W. Haigh and Henry Stonehouse, of Yorkshire College, Leeds; A. D. Roberts, Jas. Foster, and Bernard H. Woodyatt, of Owens College, Manchester; Arthur L. Reid, of St. Bartholomew's Hospital; Tom Longmore and Herbert G. Nichols, of Queen's College, Birmingham; Roger P. Wilson, of Liverpool; and James Watson, of Glasgow University.

Anatomy Only.—Federico Uzumbado, of Guy's Hospital; Sydney C. Legge and George R. Northwood, of Queen's College, Birmingham; Francis R. Gibbs, Frederick A. Cooke, and Stephen Langton, of St. Mary's Hospital: Arthur David Griffiths, of Bristol Medical School; Bruce E. Edge, Harry W. Pritchard, and Richmond Goulden, of Owens College, Manchester; John Challice, of London Hospital; T. Dobson Bell and George W. Gostling, of University College; and James E. Gordon, of Glasgow University.

Physiology Only.—Leonard Bostock and John Tait, of Owens College, Manchester; Arthur P. Woollwright and William Wyllis, of St. Bartholomew's Hospital; Percy Slack, of Sheffield Medical School, John Tilsley and Walter A. A. P. Price, of Bristol Medical School; William H. Cooper, of Bristol and Mr. Cooke's School of Anatomy and Physiology; Ambrose H. Palmer and Thomas Astbury, of Queen's College, Birmingham; Richard S. Olver, of King's College; Charles H. Nicholson, of Middlesex Hospital; and H. G. Larnder, of St. Mary's Hospital.

Passed on the 8th inst.:—

Anatomy and Physiology.—Joseph Griffiths and J. Gordon Williamson, students of Edinburgh University; Ernest Michels, of Berlin University; A. Sibert Barrow and W. Reynolds Trythall, of Middlesex Hospital; C. Marten Perry, of King's College and Edinburgh University; R. Moorson Wood, of King's College; Roland H. Fletcher, H. Pode Miles, and J. Inchbald Johnson, of University College; Frederick C. James, A. W. Viner Clarke, and William G. Stone, of St. Thomas's Hospital; J. Patrick O'Hea, Charles J. MacDonald, and Samuel E. Rigg, of St. Bartholomew's Hospital; Henry W. Beach, of Guy's Hospital.

Anatomy Only.—S. Seymour Wallis, T. Marshall Nicholson, Frank A. Evison, and J. Wallace Sweetlove, of Guy's Hospital; William Roydon and Morris N. J. Rigby, of St. Bartholomew's Hospital; Michael J. Longinotto, of St. Thomas's Hospital; Arthur H. P. Crosby and Arthur T. Davey, of Middlesex Hospital.

Physiology Only.—Gerald S. S. Marshall, of Middlesex Hospital; W. A. Bernhard Smith, of King's College; Alex. M. Cowie, of McGill College, Montreal.

And on the 9th inst.:—

Anatomy and Physiology.—Lewis Feaver, George Cawley, William J. Codrington, and Allan Kennington, of St. Bartholomew's Hospital; Joseph S. Pearse, John F. W. Waters, and Frederick W. Lewis, of Middlesex Hospital; James H. Bennett, Frederick W. Barrett, Edward

### THE STUDENT OF MEDICINE—(continued).

P. R. Tregaskis, and James C. Spillare, of London Hospital; John Phillips, of University College; Claude R. Lucas and W. Harland Peake, of Guy's Hospital; William R. Hanbury, of St. Thomas's Hospital; E. V. R. Fooks, of Charing Cross Hospital.

ANATOMY ONLY.—Vyner Graham, Wallace Pomeroy, A. Douglas Cowburn, and Savignac B. Stedman, of St. Thomas's Hospital; Thomas Rhind and Palemon Best, of University College; Lewin Bensted, W. Matthews Price, and E. Shelton Roberts, of Guy's Hospital; Toft Barker, of St. Bartholomew's Hospital; Edmund W. Gregor, of Middlesex Hospital; P. L. Watkin-Williams, of Middlesex Hospital and Mr. Cooke's School of Anatomy and Physiology; S. Mervin Meyrick, of St. Mary's Hospital.

PHYSIOLOGY ONLY.—Bertram J. Collyer, of St. Bartholomew's Hospital; Lionel T. Wells, of St. Mary's Hospital.

Passed on the 10th inst.:—

ANATOMY AND PHYSIOLOGY.—Frank Pershouse, of St. Thomas's Hospital; Robert Ballard, of St. Bartholomew's Hospital; J. Blyth Bayfield, of King's College; L. Constantine Bean, of University College; Wilfred G. Mumford, of Guy's Hospital.

ANATOMY ONLY.—L. Addams Williams, Louis J. A. de Gébert, T. H. Pryce Morris, and Hubert Williams, of Middlesex Hospital; J. Sydney Williams, of London Hospital; Archibald J. Campbell and G. Robert Harcourt, of St. Thomas's Hospital; Ernest J. Manlove, Edward W. Lee, and F. Ernest Feilden, of St. Bartholomew's Hospital; Ernest G. Annis, Henry Hewetson, and Arthur J. Mathison, of Guy's Hospital; Reginald W. Lake and A. Minter Watts, of University College; Arthur Barnes, H. Walter Whitley, and William J. Robertson, of Charing-cross Hospital; Ernest Shepherd and John Greech, of St. Mary's Hospital; Alfred W. Austen, of Westminster Hospital.

PHYSIOLOGY ONLY.—William C. Pitt and Robert Serjeant, of Guy's Hospital; Henry W. Armit, Henry A. Andrews, A. Armitage Humphrys, and Herbert C. Wimble, of St. Bartholomew's Hospital; C. Hubert Ackland, of Charing Cross Hospital; Arthur E. Syme, of Melbourne University and Mr. Cooke's School of Anatomy and Physiology; Alick C. Strand and Hobart J. W. Barlee, of Middlesex Hospital; Barnett Saul, of Charing Cross Hospital and Owens College, Manchester; Hubert A. Madge and Edgar J. Rowbotham, of Charing Cross Hospital; Samuel E. Price, of London Hospital and Mr. Cooke's School of Anatomy and Physiology; W. Shackfield Newton, of London Hospital; Courtenay C. Parsons, J. Ward Summerhays, J. E. Bishop Wells, and H. J. Kinahan Bamfield, of St. Mary's Hospital; H. Merredith Harrison, of St. Thomas's Hospital.

Passed on the 13th inst.:—

ANATOMY AND PHYSIOLOGY.—A. Kenward Matthews, Frank Cowan, and Thomas Burrow, of Guy's Hospital; Thomas O. Halliwell, and Alfred L. Fuller, of St. Thomas's Hospital; Thomas G. Wakeling, of St. Bartholomew's Hospital; Francis J. Clowes, of St. Bartholomew's Hospital and Mr. Cooke's School of Anatomy and Physiology; G. Douglas Kettlewell, of London Hospital.

ANATOMY ONLY.—E. D. J. O'Malley, of Middlesex Hospital; J. Noakes Hall and Richard D. Evans, of St. Mary's Hospital; F. Vincent Danne, of Westminster Hospital and Mr. Cook's School of Anatomy and Physiology; David Evans, of University College; William C. Hutley and A. C. Bazely Casson, St. Bartholomew's Hospital; and Ernest R. Badcock, of London Hospital.

PHYSIOLOGY ONLY.—S. Robinson Hallam, of St. Thomas's Hospital; Frank Romer, of St. George's Hospital; and Herbert Harrison, of London Hospital.

Passed on the 14th inst.:—

In ANATOMY AND PHYSIOLOGY.—Messrs. Cuthbert Henry Jones Lockyer, Charing Cross Hospital; William Douglas Knocker, St. Thomas's Hospital; William Edward Nickolls Dunn, Herbert Watson Southey, and George Alfred Harrison, St. Bartholomew's Hospital; Henry James Dean, John William Sames, and Richard Hamilton Townend, London Hospital; Lancelot Hugh Downman Hale and Edward Blackett, St. George's Hospital; and Charles Ryall, Westminster Hospital.

The following gentlemen passed in ANATOMY only:—Messrs. Harold Francis Humphreys, William Warner Henson, and Frank Henry Loveless Cloud, Guy's Hospital; William Spinks Webb and George Alfred Hayden, London Hospital; Gwilym Lewis, St. Mary's and Mr. Cook's School of Anatomy and Physiology; and John Claudius Young, Buffalo Medical University, N.S.A.

The following gentlemen passed in PHYSIOLOGY only:—Messrs. Oswald Challis, St. George's Hospital; Debonnaire Frederick Maunsell, Fred. William Waters, and Michael Aubrey Teale, St. Thomas's Hospital; Richard Withers Gilmour, St. Bartholomew's Hospital; and Sidney Hugh Langston Archer, London Hospital.

### ATHLETICS.

### Cricket.

*Westminster Hospital v. Totteridge Park.*—Played on Saturday, July 4th, at Totteridge, and resulted in a draw in favour of Westminster. Hospital: D. G. Kennard, run out, 1; G. M. Gilbert, b Drewell, 16; R. J. Stilwell b Cooke, 61; R. J. Finch, l b w Drewell, 50; W. Fleming, c and b Drewell, 8; G. Norman b Cooke, 0; J. F. Baxter, b Drewell, 2; M. Farrant, c Calvert b Drewell, 4; D Witham c and b Cooke, 1; W. B. Winckworth b Bell, 12; E. Drake, not out, 7; b, 31; l b, 5; wides, 3—39; total, 201. Totteridge Park: J. Aris, run out, 8; J. H. Bell, b Fleming, 3; Drewell, c Baxter b Finch, 37; G. Freeman, b Fleming, 5; W. Currie, c Finch b Fleming, 27; W. Cooke, b Fleming, 3; G. Calvert, st Baxter b Norman, 30; A. Mitchell, b Norman, 12; Rev. J. Bell, not out, 25; J. Prosser, not out, 1; G. Craig did not bat; b, 7; lb, 5; wides, 2—14; total, 165.

